# Investigation of generalized piezoelectric-thermoelastic problem with nonlocal effect and temperature-dependent properties

**DOI:** 10.1016/j.heliyon.2018.e00860

**Published:** 2018-10-17

**Authors:** Danni Li, Tianhu He

**Affiliations:** School of Science, Lanzhou University of Technology, Lanzhou 730050, China

**Keywords:** Applied mathematics, Mechanical engineering

## Abstract

In the generalized thermoelasticity with fractional order heat conduction and nonlocal elasticity, a generalized piezoelectric-thermoelastic problem of a both-end-fixed finite length piezoelectric rod with temperature-dependent properties and subjected to a moving heat source is investigated. The dimensionless governing equations are formulated and then solved by Laplace transform and its numerical inversion. In calculation, the effects of the nonlocal parameter, the fractional order parameter and the temperature-dependent properties on the non-dimensional temperature, displacement, stress and electrical potential are explored and demonstrated graphically. The results show that they significantly influence the peak value or magnitude of the considered physical variables.

## 1 Introduction

To eliminate the paradox in classical thermoelasticity that heat propagates in media with an infinite speed [1], the generalized thermoelastic theories have been developed, of which the first one was formulated by Lord and Shulman (L-S) [2], replacing the Fourier's law with a wave-type heat conduction law (C-V model) [3, 4], and others include the temperature-rate-dependent thermoelasticity [5], the theory of thermoelasticity without energy dissipation [6], the two-temperature generalized thermoelasticity [7], the inertia entropy thermoelasticity [8], and the thermomass thermoelasticity [9] etc.

As reviewed by Diethelm [10], the concept of the fractional derivative can be dated back to 1695 when de L'Hospital asked the famous question: ‘‘What does the derivative ${\text{d}}^{\text{n}}f/\text{d}x^{\text{n}}$ for f(x) mean if n=1/2?”. From then on, fractional calculus has been developed, which is a generalization of the commonly used integer-order differentiation and integration. In the last decade, fractional calculus has attracted a great deal of attention due to its successful applications especially in heat conduction, anomalous diffusion and viscoelasticity [11, 12, 13, 14]. Recently, the Fourier's law has been further extended into fractional ones [15, 16, 17, 18, 19, 20, 21, 22, 23] to reflect the memory-dependent effect, i.e., the instantaneous change rate of a system depends on the past state.

It can be aware that the mentioned theories were generalized basically from modifying the heat conduction equation. They may be applicable to materials or structures of relatively large sizes or scales, nevertheless, may encounter challenges in some situations as stated by Eringen [24]: Classical elasticity may fail as the external characteristic length (or time) approaches to the internal characteristic length (or time). In these cases, the classical elasticity theory should be modified into the non-classical elasticity by introducing additional material length scale parameters, of which the typical models include: nonlocal elasticity proposed by Eringen [24], gradient elasticity proposed by Aifantis [25] and modified couple stress theory [26] etc. Recently, the nonlocal effect was considered by Yu et al. [27] to study a one-dimensional generalized thermoelastic problem, and was also considered by Li et al. [28] to establish the size-dependent generalized thermoelastic diffusion theory.

As a kind of smart materials, piezoelectric materials are capable of inducing strain or generating electrical energy. When driven by an applied electric field, piezoelectric materials can convert electric energy into mechanical energy to induce strain, acting as actuator. Contrarily, when subjected to mechanical stress, piezoelectric materials can generate power, acting as generator. In most cases, piezoelectric devices serve in a thermo-elastic coupling environment. To describe the piezoelectric-thermoelastic interaction, Mindlin [29] proposed the classical couple thermopiezoelasticity theory and investigated the coupled behavior of a thermopiezoelectric plate. Chandrasekharaiah [30] generalized Mindlin's theory of thermo-piezoelectricity to account for the finite speed of propagation of thermal disturbances on the basis of the first and the second thermodynamics laws. Babaei and Chen [31] researched the time-dependent dynamic response of a piezoelectric rod. For piezoelectric devices at the sub-micron-scale using as active part in micro-/nano-electromechanical systems [32, 33, 34], mostly, they require self-powering, while, the self-powering of these devices has remained an issue and more attention needs to be paid on the analysis of the piezoelectric-thermoelastic interplay at the sub-micron-scale.

Generally speaking, material properties such as the modulus of elasticity, Poisson's ratio, the coefficient of thermal expansion and the thermal conductivity etc. are temperature-dependent, which in turn affect the thermoelastic coupling behaviors. To explore these issues, many efforts have been put into studying the dynamic responses of the problems with temperature-dependent properties for the generalized thermoelastic problems [35, 36], the generalized magneto-thermoelastic problems [37, 38], the generalized thermo-piezoelectric problems [39, 40, 41], and the generalized diffusion-thermoelastic problem [42] etc.

So far, in the existing literatures, it is hard to find investigations contributed to piezoelectric devices at the sub-micron-scale by taking nonlocal effect into consideration. In present work, we focus on investigating the dynamic response of fixed piezoelectric rods with moving heat sources at both ends. The effects of temperature dependence, non-local effects and fractional order parameter on the response of the material were also considered. The variations of the considered variables are obtained and illustrated graphically. It is hoped that the present approach may provide some theoretical guidelines in designing the piezoelectric devices at the sub-micron-scale.

## 2 Background

### 2.1 Fractional order calculus

The basic ideology is to regard a fractional derivative as the inverse operation of a fractional integral, which is usually in the Riemann–Liouville form [10].

(1) $$J_{a_{1}}^{\alpha }f\left(t\right)=\frac{1}{\text{Γ(}\alpha \text{)}}\int_{a_{1}}^{t}{\left(t-s\right)}^{\alpha -1}f\left(s\right)\text{d}s,\;t\in \left[a_{1},a_{2}\right],\;\alpha >0$$

where f(t) is integrable on the interval $\left[a_{1},a_{2}\right]$, $a_{1}$, $a_{2}$ are the upper and lower limits of time t, α is the fractional order and Γ is the Gamma function. Correspondingly, the Riemann-Liouville fractional derivative is defined as

(2) $$D_{a_{1}}^{\alpha }f\left(t\right)=D^{m}J_{a_{1}}^{m-\alpha }f\left(t\right)=\frac{{\text{d}}^{m}}{\text{d}t^{m}}\left[\int_{a_{1}}^{t}\frac{{\left(t-s\right)}^{m-\alpha -1}}{\text{Γ}\left(m-\alpha \right)}f\left(s\right)\text{d}s\right]$$

where *m* is an integer which satisfies m−1<α≤m, $D^{m}$ is the common *m*-order derivative. This concept is historically the first, however, it may encounter difficulties in satisfying the initial conditions of ‘‘realworld’’ problems. Thus, the Caputo derivative was developed [10].

(3) $$D_{a_{1}}^{\alpha }f\left(t\right)=J_{a_{1}}^{m-\alpha }D^{m}f\left(t\right)=\int_{a_{1}}^{t}K_{\alpha }\left(t-s\right)f^{\left(m\right)}\left(s\right)\text{d}s$$

With $K_{\alpha }\left(t-s\right)={\left(t-s\right)}^{m-\alpha -1}/\text{Γ(}m-\alpha )$ , It can be realized that the only inconformity between the two definitions is the sequence of fractional integral and integer-order derivative, nevertheless, which may induce big differences in practical applications.

### 2.2 Eringen nonlocal elasticity model

In Eringen's nonlocal model, the stress at a point **r** correlates not only to the strain at the point **r** but also to strains at all other points of the elastic body. The stress-strain relation has the form

(4) $$\sigma_{ij}\mathrm{(r)}=\int_{V}K\left(r,r^{\prime },\chi \right)\sigma_{ij}^{\prime }\left(r^{\prime }\right)\text{d}V\left(r^{\prime }\right)$$

(5) $${\text{σ}}_{\text{ij}}^{\prime }{\text{(}r}^{\prime }\text{)}={\lambda e}_{\text{kk}}^{\prime }{\text{(}r}^{\prime }{\text{)}\delta }_{\text{ij}}+2{\mu e}_{\text{ij}}^{\prime }\left(r^{\prime }\right)$$

(6) $$e_{ij}^{\prime }\text{(}r^{\prime }\text{)}=\frac{1}{2}\left(\frac{\partial u_{j}^{\prime }{\text{(}r}^{\prime }\text{)}}{\partial r_{\text{i}}^{\prime }}+\frac{\partial u_{\text{i}}^{\prime }{\text{(}r}^{\prime }\text{)}}{\partial r_{j}^{\prime }}\right)$$

where $\sigma_{ij}\text{(}r\text{)}$ is the nonlocal stress component, $\sigma_{ij}^{\prime }{\text{(}r}^{\prime }\text{)}$ the local stress component, $e_{ij}^{\prime }{\text{(r}}^{\prime }\text{)}$ the local strain component, λ,μ Lame's constants, $\delta_{ij}$ the Kronecker delta, $e_{kk}^{\prime }$ the local cubic dilation, $u_{i}^{\prime }{\text{(}r}^{\prime }\text{)}$ the local displacement component and *V* the volume of the body. The kernel function $K\left({r\text{,}r}^{\prime }\text{,χ}\right)$ depends on the distance $\Delta =\left\|r-r^{\prime }\right\|$ where the dimensionality of **r** as well as $r^{\prime }$ is generally 3D. The material constant χ is dimensionless and defined as χ=ea/l, where *a* is an internal characteristic length, e.g., length of C-C bond, or lattice spacing, granular distance etc., and *l* is an external characteristic length scale, e.g., wavelength, crack length, size of the sample etc. e is a non-dimensional material property for calibrating the model and ea is referred to as nonlocal parameter. Once the kernel function $K\left(r,r^{\prime },\chi \right)$ is specified as [24].

(7) $$K\left(r,r^{\prime },\chi \right)=\frac{1}{2\pi l^{2}\chi^{2}}K_{0}\left(\frac{\left\|r-r^{\prime }\right\|}{l\chi }\right)$$

Eq. (4) can be simplified as

(8) $$\left(1-{\left(ea\right)}^{2}\nabla^{2}\right)\sigma_{ij}\left(r\right)=\sigma_{ij}^{\prime }\left(r^{\prime }\right)$$

## 3 Theory

### 3.1 The basic equations and formulation of the problem

In the absence of body force and free charge, the piezoelectric-thermoelastic governing equations with nonlocal elasticity and fractional order heat conduction for linear thermo-piezoelectric media are as follows.

Motion equation

(9) $$\sigma_{ij,j}=\rho {\ddot{u}}_{i}$$

Energy equation

(10) $$\rho \left(\dot{S}T_{0}-Q\right)+q_{i,i}=0$$

Gauss equation and electric field relation

(11) $$D_{i,i}=0,\;E_{i}=-\phi_{,i}$$

Strain-displacement relations

(12) $$e_{ij}^{\prime }=\frac{1}{2}\left(u_{i,j}^{\prime }+u_{j,i}^{\prime }\right)$$

Constitutive equations

(13) $$\left(1-{\left(ea\right)}^{2}\nabla^{2}\right)\sigma_{ij}=c_{ijkl}e_{kl}^{\prime }-h_{ijk}E_{k}-\lambda_{ij}\theta$$

(14) $$D_{i}=h_{ijk}e_{jk}^{\prime }+\varepsilon_{ij}E_{j}+p_{i}\theta$$

(15) $$\rho S=\frac{\rho C_{E}}{T_{0}}\theta +\lambda_{ij}e_{ij}^{\prime }+p_{i}E_{i}$$

Eqs. (13), (14), and (15) are formulated by introducing the nonlocal elasticity into the linear thermo-piezoelectric governing equations first derived by Chandrasekharaih in [30].

The fractional order heat conduction equation advocated by Sherief et al. [16].

(16) $$q_{i}+\tau_{0}\frac{\partial^{\alpha }}{\partial t^{\alpha }}q_{i}=-\kappa_{ij}\theta_{,j}$$

In the above equations, a comma followed by a suffix denotes material derivative and a superposed dot denotes the derivative with respect to time. $D_{i}$ is the component of electric displacement, $E_{i}$ the component of electric field vector, ρ mass density, S entropy, $q_{i}$ the component of heat flux vector, Q the strength of the applied heat source per unit mass, $\;c_{ijkl}$ the elastic constants, $h_{ijk}$ the piezoelectric constants, $\lambda_{ij}$ the thermal modulus, $\varepsilon_{ij}$ the dielectric constants, $p_{i}$ the pyroelectric constant, $C_{E}$ the specific heat at constant deformation, ϕ the electric potential, $\kappa_{ij}$ the coefficient of thermal conductivity, $\theta =T-T_{0}$ the temperature increment, $T_{0}$ the initial reference temperature, T the absolute temperature, t time, $\tau_{0}$ thermal relaxation time, and α the fractional order parameter within 0<α≤1.

**Fig. 1:**
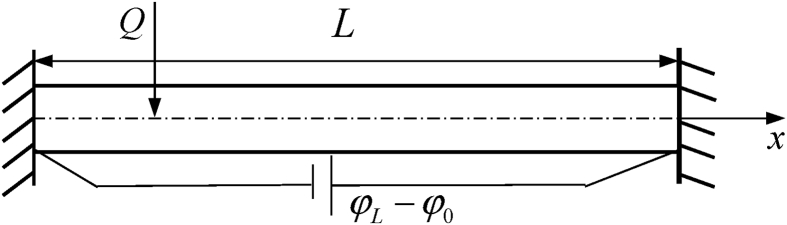
The sketch of the piezoelectric rod subjected to a moving heat source along its axis and fixed at both ends.

To apply the above equations to a concrete problem, we consider a finite length piezoelectric rod with temperature-dependent properties. The rod is fixed at both ends and subjected to a moving heat source as shown in Fig. 1. This problem can be assumed to be one-dimensional and thus all the considered variables are only functions of *x* and *t*. So that, the only non-zero displacement component is $u_{x}^{\prime }=u\left(x,t\right)$.

For this one-dimensional piezoelectric problem, it can be treated as isotropic case and the basic equations reduce accordingly to

(17) $$\sigma_{xx,x}=\rho \ddot{u}$$

(18) $$\rho \left(S_{,t}T_{0}-Q\right)+q_{x,x}=0$$

(19) $$D_{x,x}=0,\;E_{x}=-\phi_{,x}$$

(20) $$e_{xx}=u_{,x}$$

(21) $$\left(1-{\left(ea\right)}^{2}\nabla^{2}\right)\sigma_{xx}=c_{11}e_{xx}-h_{11}E_{x}-\lambda_{11}\theta$$

(22) $$D_{x}=h_{11}e_{xx}+\varepsilon_{11}E_{x}+p_{1}\theta$$

(23) $$\rho S=\frac{\rho C_{E}}{T_{0}}\theta +\lambda_{11}e_{xx}+p_{1}E_{x}$$

(24) $$q_{x}+\tau_{0}\frac{\partial^{\alpha }}{\partial t^{\alpha }}q_{x}=-\kappa_{11}\theta_{,x}$$

Substituting from (21) into (17), (22) into the first equation in (19), and (23), (24) into (18), it results in

(25) $$c_{11}\frac{\partial^{2}u}{\partial x^{2}}+h_{11}\frac{\partial^{2}\phi }{\partial x^{2}}-\lambda_{11}\frac{\partial \theta }{\partial x}=\rho \left(1-{\left(ea\right)}^{2}\nabla^{2}\right)\frac{\partial^{2}u}{\partial x^{2}}$$

(26) $$h_{11}\frac{\partial^{2}u}{\partial x^{2}}-\varepsilon_{11}\frac{\partial^{2}\phi }{\partial x^{2}}+p_{1}\frac{\partial \theta }{\partial x}=0$$

(27) $$\kappa_{11}\frac{\partial^{2}\theta }{\partial x^{2}}+\left(1+\tau_{0}\frac{\partial^{\alpha }}{\partial t^{\alpha }}\right)\left(\rho Q-\rho C_{E}\frac{\partial \theta }{\partial t}-\lambda_{11}T_{0}\frac{\partial^{2}u}{\partial x\partial t}+p_{1}T_{0}\frac{\partial^{2}\phi }{\partial x\partial t}\right)=0$$

where $\;c_{11}=\left(\lambda +2\mu \right),\lambda_{11}=\left(3\lambda +2\mu \right)\alpha_{t}$, and $\alpha_{t}$ is the coefficient of the linear thermal expansion.

As for the temperature-dependent material properties, such as the Lame's constants and the heat conductivity, they may be assumed to be

(28) $$\lambda =\lambda_{0}f\left(\theta \right),\;\mu =\mu_{0}f\left(\theta \right),\;\kappa =\kappa_{0}f\left(\theta \right)$$

where $\lambda_{0}$, $\mu_{0}$, $\kappa_{0}$ are the temperature-independent properties, and f(θ) is a function of temperature. In the study of the relation between the elastic modulus and the temperature, Rishin et al. [43] presented that

(29) $$f\left(\theta \right)=1-\alpha^{*}\theta$$

where $\alpha^{*}$ is an empirical constant. For simplification and without loss of generality, Eq. (29) can be further approximated as

(30) $$f\left(\theta \right)=1-\alpha^{*}T_{0}$$

Substituting from (28) and (30) into (25), (26), (27), we get

(31) $$c_{11}\frac{\partial^{2}u}{\partial x^{2}}+h_{11}\frac{\partial^{2}\phi }{\partial x^{2}}-\lambda_{11}\frac{\partial \theta }{\partial x}=\rho \left(1-{\left(ea\right)}^{2}\nabla^{2}\right)\frac{\partial^{2}u}{\partial x^{2}}$$

(32) $$h_{11}\frac{\partial^{2}u}{\partial x^{2}}-\varepsilon_{11}\frac{\partial^{2}\phi }{\partial x^{2}}+p_{1}\frac{\partial \theta }{\partial x}=0$$

(33) $$\kappa \frac{\partial^{2}\theta }{\partial x^{2}}+\left(1+\tau_{0}\frac{\partial^{\alpha }}{\partial t^{\alpha }}\right)\left(\rho Q-\rho C_{E}\frac{\partial \theta }{\partial t}-\lambda T_{0}\frac{\partial^{2}u}{\partial x\partial t}+p_{1}T_{0}\frac{\partial^{2}\phi }{\partial x\partial t}\right)=0$$

To normalize the governing equations, the following dimensionless quantities are introduced

(34) $$x^{*}=c_{0}\eta_{0}x,\;u^{*}=c_{0}\eta_{0}u,\;L^{*}=Lc_{0}\eta_{0},\;t^{*}=c_{0}^{2}\eta_{0}t,\;\tau_{0}^{*}=c_{0}^{2}\eta_{0}\tau_{0},\;\theta^{*}=\frac{\theta }{T_{0}},\;\sigma_{xx}^{*}=\frac{\sigma_{xx}}{c_{11}},\;D_{x}^{*}=\frac{D_{x}}{h_{11}},\;Q^{*}=\frac{Q}{\kappa T_{0}c_{0}^{2}\eta_{0}^{2}},\;c_{0}^{2}=\frac{c_{11}}{\rho },\;\eta_{0}=\frac{\rho C_{E}}{\kappa },\phi^{*}=\frac{\varepsilon_{11}}{h_{11}L}\phi \;{\left(ea\right)}^{*}=\;c_{0}\eta_{0}\left(ea\right)$$

After normalization, the governing equations take the forms (dropping all the asterisks at the upper right corner for convenience hereafter)

(35) $$\frac{\partial^{2}u}{\partial x^{2}}+g_{1}\beta_{2}\frac{\partial^{2}\phi }{\partial x^{2}}-g_{2}\frac{\partial \theta }{\partial x}=\beta_{2}\left(1-{\left(ea\right)}^{2}\frac{\partial^{2}}{\partial x^{2}}\right)\frac{\partial^{2}u}{\partial t^{2}}$$

(36) $$\frac{\partial^{2}u}{\partial x^{2}}-g_{3}\frac{\partial^{2}\phi }{\partial x^{2}}+f_{1}\frac{\partial \theta }{\partial x}=0$$

(37) $$\frac{\partial^{2}\theta }{\partial x^{2}}+\left(1+\tau_{0}\frac{\partial^{\alpha }}{\partial t^{\alpha }}\right)\left(\rho Q-\frac{\partial \theta }{\partial t}-f_{2}\frac{\partial^{2}u}{\partial x\partial t}+\beta_{2}f_{3}\frac{\partial^{2}\phi }{\partial x\partial t}\right)=0$$

in which

(38) $$g_{1}=\frac{c_{0}\eta_{0}Lh_{11}^{2}}{\varepsilon_{11}c_{11}},{\;\text{g}}_{2}=\frac{\lambda_{11}T_{0}}{c_{11}},{\;\text{g}}_{3}=c_{0}\eta_{0}L,\;\;f_{1}=\frac{p_{1}T_{0}}{h_{11}},\;f_{2}=\frac{\lambda_{11}}{\rho c_{E}},\;f_{3}=\frac{p_{1}c_{0}Lh_{11}}{\kappa \varepsilon_{11}},\beta_{2}=\frac{1}{1-\alpha^{*}T_{0}}$$

The initial conditions to supplement the governing equations are

(39) $$u\left(x,0\right)=\dot{u}\left(x,0\right)=0,\;\;\theta \text{(}x,0\text{)}=\dot{\theta }\text{(}x,0\text{)}=\text{0,}\;\;\phi \text{(}x,0\text{)}=\dot{\phi }\text{(}x,0\text{)}=\text{0}$$

and the boundary conditions are

(40) $$u\left(0,t\right)=u\left(L,0\right)=0,\;\phi \text{(0,}t\text{)}=\phi \text{(}L\text{,}t\text{)}=\text{0,}\;\frac{\partial \theta \left(0,t\right)}{\partial x}=\frac{\partial \theta \left(L,t\right)}{\partial x}=0$$

The moving heat source is in the non-dimensional form as

(41) $$Q=Q_{0}\delta \left(x-vt\right)$$

where $Q_{0}$ is a constant, v is the velocity and δ is the Dirac function.

### 3.2 Solutions in the Laplace domain

Applying the Laplace transform defined by

(42) $$L\left[f\left(t\right)\right]=\bar{f}\left(s\right)=\int_{0}^{\infty }e^{-st}f\left(t\right)dt$$

to Eqs. (35), (36), and (37), we obtain

(43) $$g_{4}\left[\left({1+\beta_{2}\left(ea\right)}^{2}s^{2}\right)\frac{{\text{d}}^{2}}{\text{d}x^{2}}-\beta_{2}s^{2}\right]\bar{u}+g_{1}\beta_{2}\frac{{\text{d}}^{2}\bar{\phi }}{\text{d}x^{2}}-g_{2}\frac{\text{d}\bar{\theta }}{\text{d}x}=0$$

(44) $$\frac{{\text{d}}^{2}\bar{u}}{\text{d}x^{2}}-g_{3}\frac{{\text{d}}^{2}\bar{\phi }}{\text{d}x^{2}}+f_{1}\frac{\text{d}\bar{\theta }}{\text{d}x}=0$$

(45) $$\left(\frac{{\text{d}}^{2}}{\text{d}x^{2}}-s+\tau_{0}s^{\alpha +1}\right)\bar{\theta }-f_{2}s\left(1+\tau_{0}s^{\alpha }\right)\frac{\text{d}\bar{u}}{\text{d}x}+f_{3}\beta_{2}s\left(1+\tau_{0}s^{\alpha }\right)\frac{\text{d}\bar{\phi }}{\text{d}x}=-\left(1+\tau_{0}s^{\alpha }\right)f_{4}{\text{e}}^{-\frac{s}{v}x}$$

where $g_{4}=\rho c_{0}^{2}/c,\;\;f_{4}=\rho Q_{0}/v$.

Eliminating $\;\bar{\theta }\;$ and $\;\bar{\phi }\;$ from Eqs. (43), (44), and (45), it can be arrived at the equation satisfied by $\bar{u}$ as

(46) $$m_{1}\frac{{\text{d}}^{4}\bar{u}}{\text{d}x^{4}}+m_{2}\frac{{\text{d}}^{2}\bar{u}}{\text{d}x^{2}}+m_{3}\bar{u}=m{\text{e}}^{-\frac{s}{v}x}$$

where

(47) $$\begin{array}{l} m_{1}=\frac{{-f_{1}g_{4}g_{3}\text{(}1+\beta_{2}\text{(}ea\text{)}}^{2}s^{2}\text{)}-g_{1}\beta_{2}f_{1}}{s\text{(}1+\tau_{0}s^{\alpha }\text{)}\text{(}g_{1}\beta_{2}f_{1}-g_{2}g_{3}\text{)}}\\ m_{2}=1-f_{1}\text{(}\frac{g_{4}\beta_{2}s}{g_{2}\text{(}1+\tau_{0}s\text{)}}+f_{2}\text{)}+\frac{g_{1}f_{1}^{2}g_{4}\beta_{2}^{2}s}{g_{2}\text{(}1+\tau_{0}s^{\alpha }\text{)}\text{(}g_{1}\beta_{2}f_{1}-g_{2}g_{3}\text{)}}\\ \;\;\;\;\;\;\;-\text{(}\beta_{2}f_{1}f_{3}-g_{3}\text{)}\frac{{f_{1}g_{4}\text{(}1+\beta_{2}\text{)}ea\text{)}}^{2}s^{2}\text{(}+g_{2}}{g_{1}\beta_{2}f_{1}-g_{2}g_{3}}\\ m_{3}=\frac{\text{(}\beta_{2}f_{1}f_{3}-g_{3}\text{)}f_{1}\beta_{2}}{g_{1}\beta_{2}f_{1}-g_{2}g_{3}}g_{4}s^{2}\\ m=\frac{f_{1}f_{4}}{v} \end{array}$$

The solution for $\bar{u}$ has the form

(48) $$\bar{u}=C_{1}{\text{e}}^{\lambda_{1}x}+C_{2}{\text{e}}^{\lambda_{2}x}+C_{3}{\text{e}}^{\lambda_{3}x}+C_{4}{\text{e}}^{\lambda_{4}x}+K{\text{e}}^{-\frac{s}{v}x}$$

where $C_{i}\;\left(i=1-4\right)$ are undetermined parameters, and ${{K=m/[m_{1}\left(s/v\right)}^{4}+m_{2}\left(s/v\right)}^{2}+m_{3}]$. $\lambda_{i}\;\left(i=1-4\right)$ are the roots of the following characteristic equation

(49) $$\lambda^{4}+\frac{m_{2}}{m_{1}}\lambda^{2}+\frac{m_{3}}{m_{1}}=0$$

which take

(50) $$\lambda_{1}=-\lambda_{2}=\sqrt{\frac{-m_{2}+{\sqrt{m_{2}^{2}-4m_{1}m}}_{3}}{2m_{1}}},\lambda_{3}=-\lambda_{4}=\sqrt{\frac{-m_{2}-{\sqrt{m_{2}^{2}-4m_{1}m}}_{3}}{2m_{1}}}$$

Similarly, we can find the solutions for $\bar{\phi }$ and $\bar{\theta }$ respectively, as

(51) $$\bar{\phi }=a_{1}C_{1}{\text{e}}^{\lambda_{1}x}+a_{2}C_{2}{\text{e}}^{\lambda_{2}x}+a_{3}C_{3}{\text{e}}^{\lambda_{3}x}+a_{4}C_{4}{\text{e}}^{\lambda_{4}x}+a_{5}K{\text{e}}^{-\frac{s}{v}x}+D_{1}x+D_{2}$$

(52) $$\bar{\theta }=b_{1}C_{1}{\text{e}}^{\lambda_{1}x}+b_{2}C_{2}{\text{e}}^{\lambda_{2}x}+b_{3}C_{3}{\text{e}}^{\lambda_{3}x}+b_{4}C_{4}{\text{e}}^{\lambda_{4}x}+b_{5}K{\text{e}}^{-\frac{s}{v}x}+\frac{g_{3}}{f_{1}}D_{1}x+D_{3}$$

where $D_{i}\left(i=1-3\right)$ are undetermined parameters and

(53) $$\begin{array}{l} a_{1}=a_{2}=\frac{g_{4}f_{1}}{g_{1}\beta_{2}f_{1}-g_{2}g_{3}}\left\{\frac{\beta_{2}s^{2}}{\lambda_{1}^{2}}-\left[{g_{4}\text{(}1+\beta_{2}\text{(}ea\text{)}}^{2}s^{2}\text{)}+\frac{g_{2}}{f_{1}}\right]\right\}\\ a_{3}=a_{4}=\frac{g_{4}f_{1}}{g_{1}\beta_{2}f_{1}-g_{2}g_{3}}\left\{\frac{\beta_{2}s^{2}}{\lambda_{3}^{2}}-\left[{g_{4}\text{(}1+\beta_{2}\text{(}ea\text{)}}^{2}s^{2}\text{)}+\frac{g_{2}}{f_{1}}\right]\right\}\\ a_{5}=\frac{f_{1}g_{4}}{g_{1}f_{1}\beta_{2}-g_{2}g_{3}}\left[{\beta_{2}v^{2}-g_{4}\text{(}1+\beta_{2}\text{(}ea\text{)}}^{2}s^{2}\text{)}-\frac{g_{2}}{f_{1}}\right]\\ b_{1}=-b_{2}=\text{(}\frac{g_{3}}{f_{1}}a_{1}-\frac{1}{f_{1}}\text{)}\lambda_{1}\;\;\;\;b_{3}=-b_{4}=\text{(}\frac{g_{3}}{f_{1}}a_{3}-\frac{1}{f_{1}}\text{)}\lambda_{3}\\ b_{5}=-\text{(}\frac{g_{3}}{f_{1}}a_{5}-\frac{1}{f_{1}}\text{)}\frac{s}{v} \end{array}$$

Substituting from (48), (51) and (52) into (45), we obtain

(54) $$\frac{\beta_{2}f_{1}f_{3}-g_{3}}{f_{1}}D_{1}-D_{3}=0$$

By introducing the solutions for $\bar{u}$, $\bar{\theta }$ and $\bar{\phi }$ into Eq. (21), from Eqs. (43), (44), and (45), it can be obtained that

(55) $${\bar{\sigma }}_{xx}=C_{1}\left[\lambda_{1}\left(1+a_{1}j_{1}\right)-j_{2}b_{1}\right]e^{\lambda_{1}x}+C_{2}\left[\lambda_{2}\left(1+a_{2}j_{1}\right)-j_{2}b_{2}\right]e^{\lambda_{2}x}+C_{3}\left[\lambda_{3}\left(1+a_{3}j_{1}\right)-j_{2}b_{3}\right]e^{\lambda_{3}x}+C_{4}\left[\lambda_{4}\left(1+a_{4}j_{1}\right)-j_{2}b_{4}\right]e^{\lambda_{4}x}+K\left[-s\left(1+a_{5}j_{1}\right)/v-j_{2}b_{5}\right]e^{-s/vx}+D_{1}\left[j_{1}-j_{2}g_{3}/f_{1}\right]-j_{2}D_{3}$$

in which ${{j_{1}=g_{1}\beta_{2}/\text{(}1+\beta_{2}\text{(}ea\text{)}}^{2}s^{2}\text{)},\;\;j_{2}=g_{2}/\text{(}1+\beta_{2}\text{(}ea\text{)}}^{2}s^{2}\text{)}$.

To determine $C_{i}$ and $D_{i}$, the boundary conditions need to be applied to getting the following equations

(56) $$C_{1}+C_{2}+C_{3}+C_{4}+K=0$$

(57) $$C_{1}{\text{e}}^{\lambda_{1}Lc_{0}\eta_{0}}+C_{2}{\text{e}}^{\lambda_{2}Lc_{0}\eta_{0}}+C_{3}{\text{e}}^{\lambda_{3}Lc_{0}\eta_{0}}+C_{4}{\text{e}}^{\lambda_{4}Lc_{0}\eta_{0}}+K{\text{e}}^{-\left(s/v\right)Lc_{0}\eta_{0}}=0$$

(58) $$a_{1}C_{1}+a_{2}C_{2}+a_{3}C_{3}+a_{4}C_{4}+a_{5}K+D_{2}=0$$

(59) $$a_{1}C_{1}{\text{e}}^{\lambda_{1}Lc_{0}\eta_{0}}+a_{2}C_{2}{\text{e}}^{\lambda_{2}Lc_{0}\eta_{0}}+a_{3}C_{3}{\text{e}}^{\lambda_{3}Lc_{0}\eta_{0}}+a_{4}C_{4}{\text{e}}^{\lambda_{4}Lc_{0}\eta_{0}}+a_{5}K{\text{e}}^{-\left(s/v\right)Lc_{0}\eta_{0}}+D_{1}Lc_{0}\eta_{0}+D_{2}=0$$

(60) $$b_{1}C_{1}\lambda_{1}+b_{2}C_{2}\lambda_{2}+b_{3}C_{3}\lambda_{3}+b_{4}C_{4}\lambda_{4}-b_{5}K\left(s/v\right)=0$$

(61) b1C1λ1eλ1Lc0η0+b2C2λ2eλ2Lc0η0+b3C3λ3eλ3Lc0η0+b4C4λ4eλ4Lc0η0−b5K(s/v)e−(sv)Lc0η0=0

From (54) and (56), (57), (58), (59), (60), (61), all the undetermined parameters can be obtained by solving the corresponding set of equations in MATLAB software. Due to the lengthy expressions of the parameters, they are not listed here.

### 3.3 Numerical Laplace inversion

In view of the complex expressions of $\bar{u}$, $\bar{\theta }$ and $\bar{\phi }$, it is not doable for them to be inverted analytically from Laplace domain to time domain. Alternatively, they may be inverted numerically by Riemann-sum approximation method. In this method, any function $\bar{f}\left(x,s\right)$ in Laplace domain can be inverted into time domain by the formula [44].

(62) f(x,t)=eβtt[12f¯(x,β)+Re∑n=1Nf¯(x,β+inπt)(−1)n]

where Re is the real part and i is the imaginary number unit. For faster convergence, the value of β should satisfy βt≈4.7 [45].

## 4 Results and discussion

Numerical calculations are carried out to illustrate the distributions of the non-dimensional temperature, displacement, stress as well as electric potential in the piezoelectric rod, especially, the influences of the variable parameters, i.e., the nonlocal parameter, the fractional order parameter and the temperature-dependent properties, on the distributions are emphatically examined. The properties of the piezoelectric material in the case of temperature-independence are given as follows

$$c_{11}=74.1\times 10^{9}{\text{Nm}}^{-2}\;\lambda_{11}=0.621\times 10^{6}{\text{NK}}^{-1}{\text{m}}^{-2}\;\rho =7600{\text{kgm}}^{-3}\;h_{11}=0.2{\text{Cm}}^{-2}\varepsilon_{11}=0.392\times 10^{-10}{\text{Fm}}^{-1}\;C_{E}=420{\text{JKg}}^{-1}{\text{K}}^{-1}\;p_{1}=4\times 10^{-4}{\text{CK}}^{-1}{\text{m}}^{-2}\;\kappa =1.4{\text{WK}}^{-1}{\text{m}}^{-1}T_{0}=293\text{K}\;Q_{0}=10\;\tau_{0}=0.05\;L=10$$

First, to validate our results, we compare our results numerically not experimentally with those obtained in [31] by model degeneration due to the fact that it is not available to find an appropriate experimental work in the existing literatures.

**Fig. 2:**
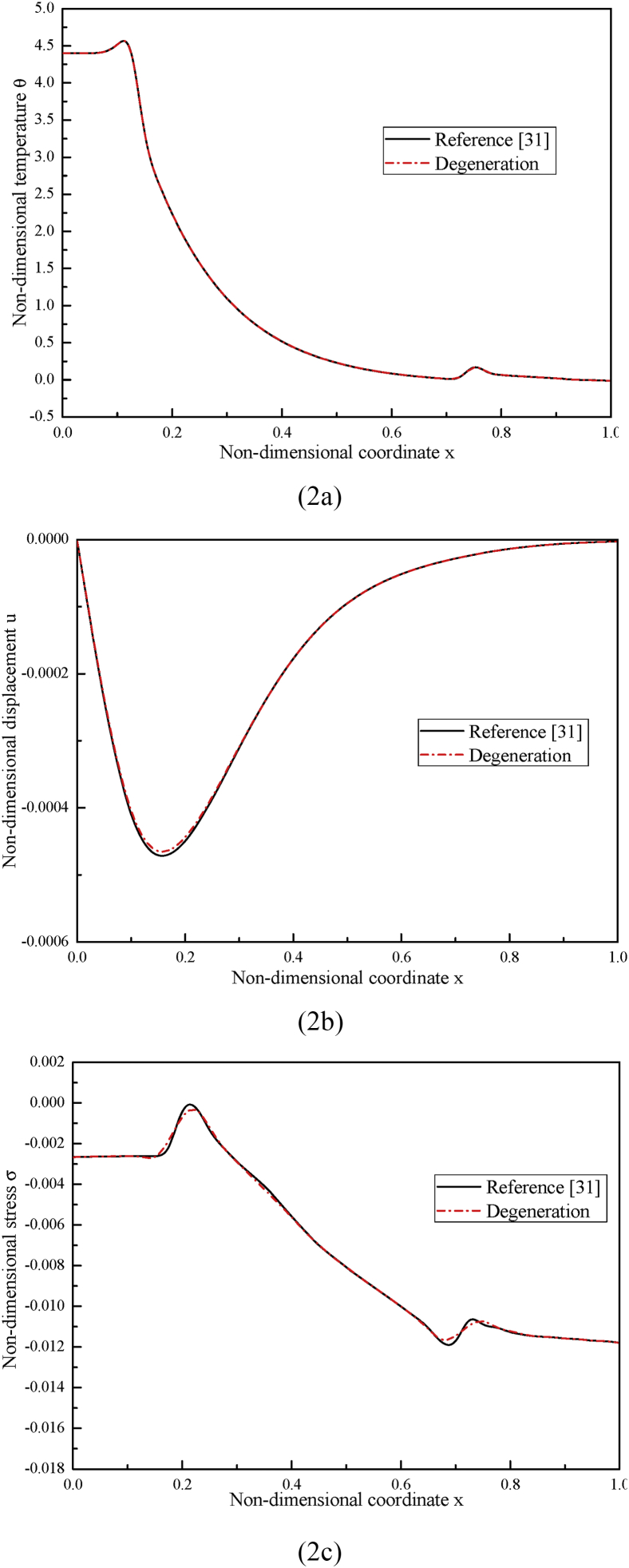
Comparisons of the distributions of (2a) the non-dimensional temperature, (2b) the non-dimensional displacement and (2c) the non-dimensional stress between the present work and reference [35] by degeneration.

Once the nonlocal parameter *ea*, the fractional-order parameter α, and the temperature-dependent factor $\beta_{2}$ are all set as 0, our model degenerates into the same model in [31]. In Fig. 2, we compare the results of the non-dimensional temperature, the displacement as well as the stress obtained from the degenerated model with those obtained at the same time *t* = 0.1848 in [31], and find that they agree with each other very well.

Next, we examine the influence of variable parameters respectively in three separate cases. In calculation, the time *t* and the velocity of the moving heat source are specified as *t* = 0.1 and v=2.

(1) Case one

**Fig. 3:**
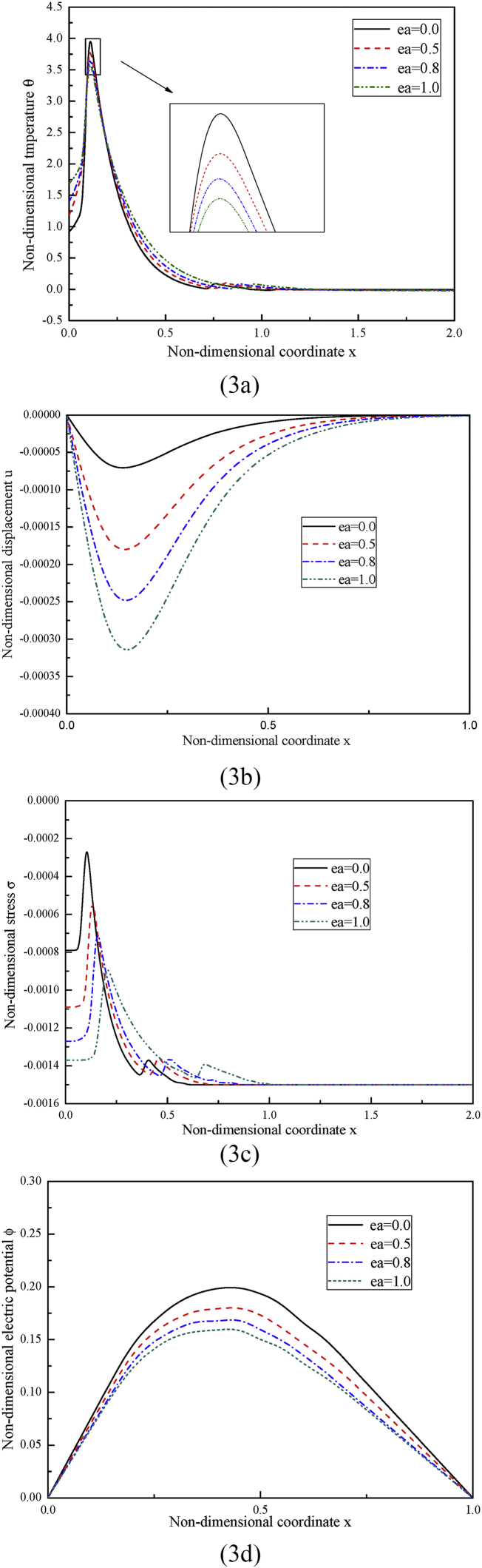
The distributions of (3a) the non-dimensional temperature, (3b) the non-dimensional displacement, (3c) the non-dimensional stress and (3d) the non-dimensional electric potential under different nonlocal parameter ea while α=1, $\beta_{2}=1$.

In case one, we examine how the nonlocal parameter ea influences the distributions of the considered variables. In calculation, four different values for ea are set, i.e., ea= 0, 0.5, 0.8, 1, of them, ea= 0 stands for the case that no nonlocal effect is included. The fractional order parameter and the temperature-dependent factor are kept as α=1 and $\beta_{2}=1$ respectively. The obtained results are illustrated in Fig. 3.

In Fig. 3(a), the non-dimensional temperature first goes up, then reaches the peak value, afterwards goes down till to zero. Due to the finite velocity of heat propagation, the non-zero values of the non-dimensional temperature are within a bounded region, which indicates that the rod beyond this region is thermally undisturbed. From Eq. (41), it can be known that the heat source moves along the rod with a constant velocity v. Once time *t* is prescribed, the distance that the heat source moves across is x=vt. At location x=vt, the heat source releases its maximum energy, which thus results in a peak value in the distribution of temperature. The nonlocal parameter ea significantly influences the variation of the non-dimensional temperature, especially the peak value, which decreases with the increase of the nonlocal parameter ea. Before the peak value, the temperature decreases with the increase of ea, while after the peak value, the temperature increases with the increase of ea. It can be observed that the curve of the temperature tends to become flatter with the increase of ea.

In Fig. 3(b), due to the thermal expansion and the fixed boundaries, the induced non-dimensional displacement is negative and the values at both ends are always zero. The variation of the non-dimensional displacement is affected remarkably by the nonlocal parameter ea and the absolute values of the non-dimensional displacement increase with the increase of the nonlocal parameter ea. For each curve, the value first goes down and then goes up.

In Fig. 3(c), the non-dimensional stress in the rod is compressive, which is due to the fact that the rod is confined not to elongate freely when it undergoes thermal expansion deformation. The nonlocal parameter ea has marked effect on the variation of the non-dimensional stress and its peak value decreases with the increase of the nonlocal parameter. For each curve, it first goes up, reaches the peak and then goes down till to zero.

In Fig. 3(c), because of the constrained thermal expansion deformation induced by the applied heat source, the rod in between the two fixed ends suffers from compressive stress, which generates electric potential inside the rod, induced by the so called the direct piezoelectric effect. As seen, the magnitude of the electric potential decreases as the non-local parameter ea increases.

(2) Case two

**Fig. 4:**
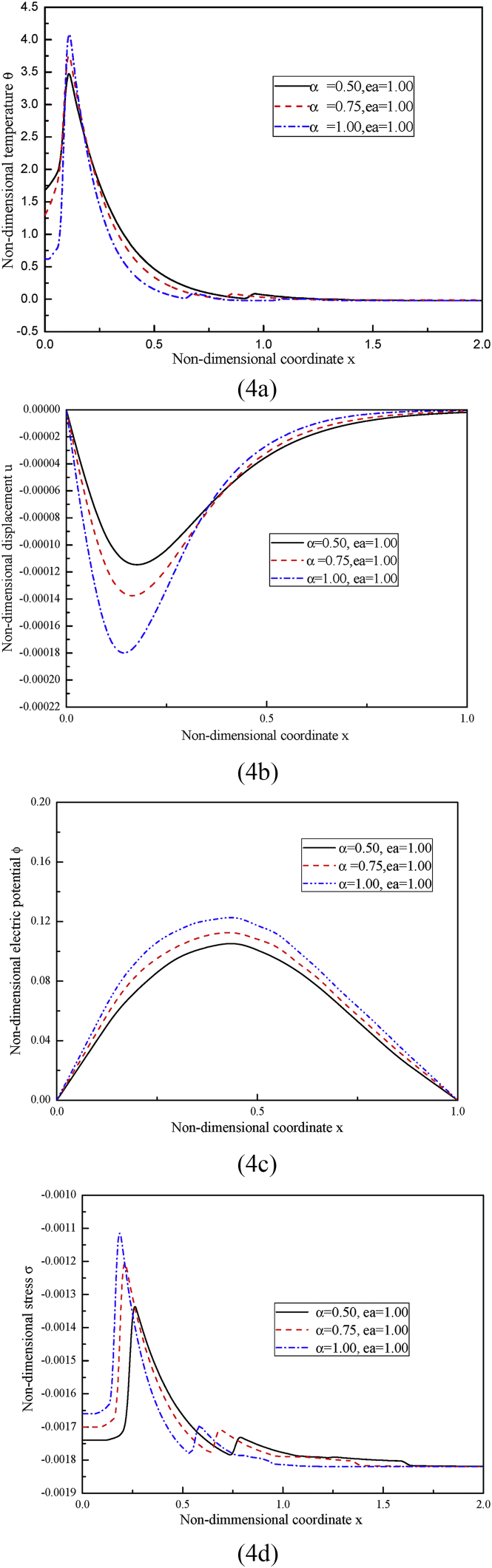
The distributions of (4a) the non-dimensional temperature, (4b) the non-dimensional displacement, (4c) the non-dimensional electric potential and (4d) the non-dimensional stress under different fractional order parameter α while ea=1, $\beta_{2}=0.8$.

In case two, we study how the fractional order parameter α affects the distributions of the considered variables. According to Sherief et al. [16], the fractional order parameter lies in the range 0<α≤1, so, three different values for α are taken, i.e., α= 0.5, 0.75, 1, of them, α= 1 corresponds to the C-V wave-type heat conduction. The nonlocal parameter and the temperature-dependent factor are kept as ea=1 and $\beta_{2}=0.8$ respectively. The obtained results are illustrated in Fig. 4.

As shown in Fig. 4, the variations of the non-dimensional temperature, displacement, electric potential and stress are all sensitive to the change of the fractional order parameter α.

In Fig. 4(a), the temperature first goes up, then reaches the peak value and subsequently goes down to zero. As seen, the curve of the temperature becomes steeper as the fractional order parameter α gets greater. The peak value of the temperature increases with the increase of the fractional order parameter.

In Fig. 4(b), the peak value magnitude of the negative displacement becomes larger as the fractional order parameter α gets larger. The curve of the displacement becomes steeper under bigger fractional order parameter α.

In Fig. 4(c), the induced electric potential increases with the increase of the fractional order parameter α.

In Fig. 4(d), the peak value magnitude of the compressive stress increases with the increase of the fractional order parameter α. The larger the fractional order parameter α is, the steeper the displacement curve is.

(3) Case three

**Fig. 5:**
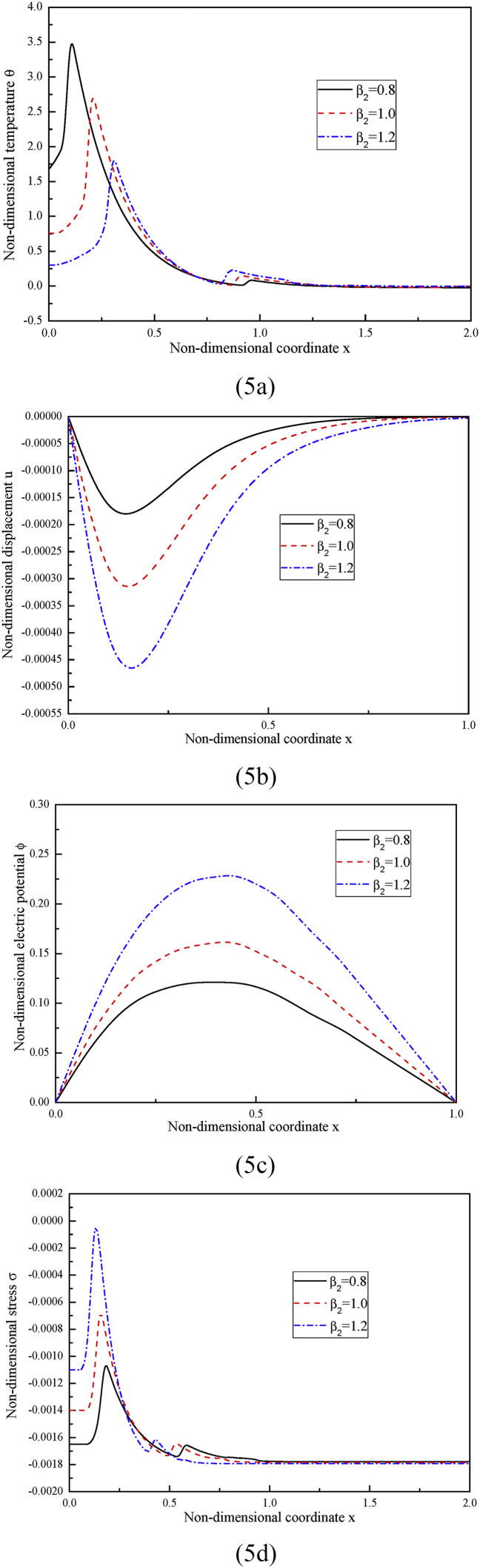
The distributions of (5a) the non-dimensional temperature, (5b) the non-dimensional displacement, (5c) the non-dimensional electric potential and (5d) the non-dimensional stress under different temperature-dependent factor $\beta_{2}$ while ea=1, α=1.

In case three, we investigate how the temperature-dependent factor $\beta_{2}$ affects the distributions of the considered variables. In calculation, three different values for $\beta_{2}$ are set, i.e., $\beta_{2}=$ 0.8, 1, 1.2, of them, $\beta_{2}=$ 1 represents that the material properties are temperature-independent. The nonlocal parameter and the fractional order parameter are kept as ea=1 and α=1 respectively. The obtained results are illustrated in Fig. 5.

As observed in Fig. 5, the distributions of all the considered variables are greatly influenced by the temperature-dependent material properties, which are related to$\beta_{2}$. From the relations in between $\;\beta_{2}=1\text{/}\left(1-\alpha^{*}T_{0}\right)$ in (38) and the expressions in (30), it can be realized that the temperature-dependent properties become smaller as $\beta_{2}$ becomes larger. Being aware of this, it can be deduced from Fig. 5(a) that the peak value of the non-dimensional temperature decrease with the decrease of the temperature-dependent properties. In Fig. 5(b) and (c), the magnitudes of the non-dimensional displacement and the non-dimensional electric potential increase as temperature-dependent properties decrease. In Fig. 5(d), the peak value of the non-dimensional stress increase as the decrease of the temperature-dependent properties decrease.

## 5 Conclusions

In the generalized thermoelasticity with fractional order heat conduction and nonlocal elasticity, a generalized piezoelectric-thermoelastic problem of a finite length piezoelectric rod with temperature-dependent properties is investigated. The rod is fixed at both ends and subjected to a moving heat source. The effects of the nonlocal parameter, the fractional order parameter and the temperature-dependent properties on the variations of the considered physical variables are examined and illustrated. From the obtained results, it can be concluded that

(1) The nonlocal parameter ea significantly influences the variations of all the considered variables. The peak values of the non-dimensional temperature and the non-dimensional stress decreases with the increases of the nonlocal parameter, the absolute values of the non-dimensional displacement increases with the increases of the nonlocal parameter and the magnitude of the electric potential decreases as the non-local parameter increases.
(2) The variations of the considered variables are all sensitive to the change of the fractional order parameter α. The peak values of all the considered variables increase with the increase of the fractional order parameter.
(3) The peak value of the non-dimensional temperature decrease with the decrease of the temperature-dependent properties, the magnitudes of the non-dimensional displacement and the non-dimensional electric potential increase with the decrease of the temperature-dependent properties, and the peak value of the non-dimensional stress increase with the decrease of the temperature-dependent properties.

## Declarations

### Author contribution statement

Danni Li: Analyzed and interpreted the data; Contributed analysis tools or data.

Tianhu He: Analyzed and interpreted the data; Contributed analysis tools or data; Wrote the paper.

### Funding statement

This work was supported by National Natural Science Foundation of China (11372123).

### Competing interest statement

The authors declare no conflict of interest.

### Additional information

No additional information is available for this paper.
